# Radiographic evaluation of a cross-shaped incision technique for thick-gingiva and thin-gingiva patients treated with implant-supported fixed prosthesis

**DOI:** 10.1186/s12903-021-02019-8

**Published:** 2021-12-18

**Authors:** Wen Luo, Xinyu Wang, Yaqian Chen, Yuping Hong, Yili Qu, Yi Man, Yingying Wu

**Affiliations:** 1 State Key Laboratory of Oral Diseases and National Clinical Research Center for Oral Diseases, West China Hospital of Stomatology, Sichuan University, No.14, Sec.3, Renminnan Road, Chengdu, 610041 Sichuan China; 2 Department of Stomatology, The First Affiliated Hospital of Hainan Medical University, Haikou, 570102 China

**Keywords:** Cross-shaped incision, Gingiva, Recession of marginal gingiva, Implant, Fixed prosthesis

## Abstract

**Background:**

To evaluate a cross-shaped incision technique for thick-gingiva and thin-gingiva patients treated with implant-supported fixed prosthesis.

**Methods:**

Total 55 patients receiving cross-shaped incision were assigned into thick-gingiva group (29 cases) and thin-gingiva group (26 cases). Follow-up was performed at 3 and 12-month after final restoration.

**Results:**

Mesial and distal papilla height was significantly greater in thick-gingiva group than thin-gingiva group at 3 and 12 months, while periodontal depth and crestal marginal bone level around implant had no significant difference between the two groups during follow-up. No case of recession of buccal marginal gingiva was observed in thick-gingiva group. However, the recession of marginal gingiva of buccal aspect of the crown was found in 5 patients (19.2%) with thin-gingiva.

**Conclusions:**

The cross-shaped incision may be applied to reconstruct gingival papillae and avoid the gingival recession in patients with thick-gingiva phenotype.

*Trial registration* This study was registered at ClinicalTrials.gov (registration number NCT04706078, date 12 January 2021, Retrospectively registered).

## Background

Dental implant has been successfully used to restore missing teeth. However, food impaction at the implant site is considered as a common complication in patients with implant prostheses. Food impaction is the phenomenon in the chewing when the food dregs or fibers are pushed into the clearance by occlusal force or owing to the gingival shrinkage [1]. Food impaction includes vertical (the forceful wedging of food into the interproximal space by masticatory pressure) and horizontal (the forcing of food interproximally by tongue or cheek pressure) food impaction [2].

The loss of interproximal contact between fixed implant prostheses and adjacent teeth is the main issue of vertical food impaction, which can be solved through occlusal adjustment, re-making of the prosthesis, inlay-crown or full crown of adjacent teeth [3]. Horizontal food impaction is mostly caused by the absence of interproximal papilla, which leads to the abnormal space under the proximal connection [1, 4, 5]. Sometimes gingival incising (such as a cross-shaped incision) is necessary to reduce the soft tissue resistance to seat the restoration. Surgical injury may lead to gingival recession and the absence of interproximal papilla. Gingival phenotype, divided into thin or thick gingiva, affects the dimension of the periodontal tissue. A thick phenotype is prone to pocket formation, while a thin phenotype is prone to gingival recession following mechanical or surgical manipulation [6]. The regeneration or reconstruction of gingival papillae is a challenge to clinicians.

Various treatment plans and techniques have been proposed to restore the deficient papilla. Hard tissue augmentation during implant placement was suggested as an effective method to obtain desired inter-dental/inter-implant papillae [7]. Orthodontic procedures are proposed to enhance hard tissue profiles and improve the papillae height [8]. Both surgical and orthodontic management is designed to create the papillae in the presurgical or surgical phase. If there is a deficit of papillae during the stage-two surgery, then soft tissue grafting or vascularized interposition periosteal-connective tissue (VIP-CT) flap was recommended [9–11]. However, the management would be a great challenge to clinicians in posterior position. Therefore, a provisional restoration with proper emergence profile has been widely accepted as the treatment to reconstruct papillae around implant, although the bone may recede more from composite resin than a titanium surface [12]. In addition, the pressure against the peri-implant soft tissue may cause discomfort to patients [7]. Therefore, the cross-shaped incision across gingival sulcus could be considered as an effective and simple way for reducing the soft tissue resistance to seat the restoration with implant-supported fixed prosthesis. The cross-shaped incisions went directly to the bone surface. The length of the cross-shaped incisions was 1–2 mm in keratinaized gingiva.

This study aimed to investigate whether different gingival phenotypes have the same ability to recover from surgical injury, and we evaluated a cross-shaped incision technique for thick-gingiva and thin-gingiva patients treated with implant-supported fixed prosthesis.

## Methods

### Study design

The present study was performed in accordance with the World Medical Association Declaration of Helsinki and was approved by Ethics Committee of West China Hospital of Stomatology, Sichuan University, China (Approval No. 2009033). The study adheres to CONSORT guidelines and is registered in the ClinicalTrials.gov (registration number NCT04706078, date 12 January 2021, https://clinicaltrials.gov/ct2/show/NCT04706078). All the patients signed informed consent before the implant surgery.

### Inclusion criteria

1. Good general health, no chronic systemic diseases.
2. All subjects included in this study needed to have one missing premolar or molar teeth with adjacent natural teeth.
3. All subjects included in this study had been treated with one bone-level implant insertion in the premolar or molar region. The patients had insufficient gingival papilla height (referred to contralateral natural tooth which also had insufficient gingival papilla height) and at least 2 mm of keratinized tissue width around the implant.

### Exclusion criteria

1. Active periodontal infections.
2. Heavy smoking (> 10 cigarettes per day).

### Samples and groups

Fifty-five subjects were selected from the patients who need treatment with a cross-shaped incision technique of the Department of Oral Implantology, West China Hospital of Stomatology, Sichuan University in China between June 2018 and June 2020.

**Fig. 1:**
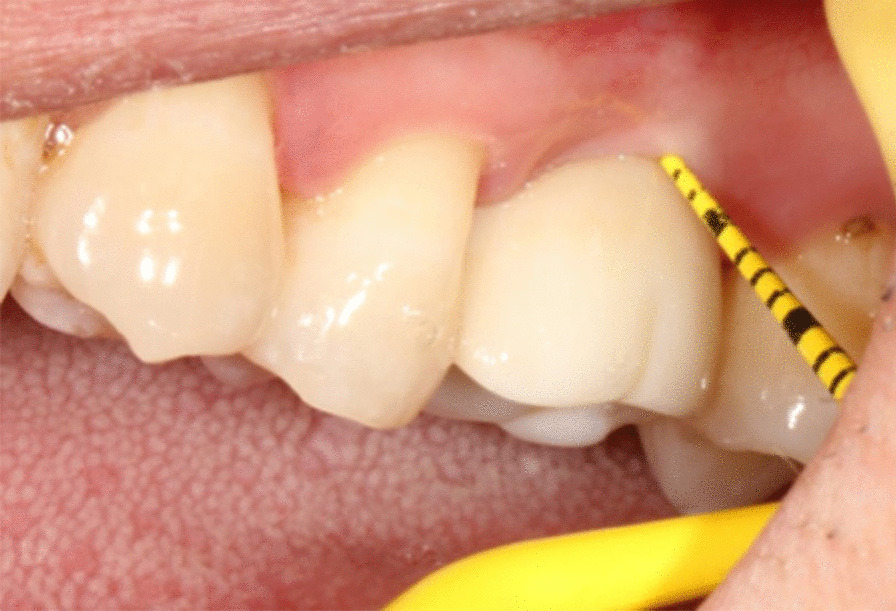
The phenotype of gingiva determined by a periodontal probe

Patients were divided into two groups according to the gingival phenotype determined by a periodontal probe. After the insertion of the probe into the facial aspect of the sulcus through the gingival margin, the simple visual method is based on the transparency of the periodontal probe through the gingival margin while probing the buccal sulcus at the midfacial aspect of the tooth. When the outline of the underlying periodontal probe can be seen through the gingival, the gingival phenotype is considered thin. The gingival phenotype is thick in the other case. When the crown shape protrusion is not obvious, the main direction of the probe is parallel to the long axis of the tooth. The peri-implant phenotype was categorized as thin-gingiva (26 cases, outline of the probe can be seen through the gingiva) or thick-gingiva (29 cases, outline of the probe cannot be seen through the gingiva) (Fig. 1) [13].

### Procedures

The bone-level implants were placed 1 mm below the alveolar bone level according to the manuals. The trial started at 3–6 months after the one-stage surgery. Impression was taken for the fabrication of definitive crown after 3-6 months of healing. Titanium abutments with the lowest gingival height (1–1.5 mm) were selected, and all of the crowns were made of zirconia. Platform switching which referred to the use of a smaller diameter abutment on a larger diameter implant collar was used in all the implants.

**Fig. 2:**
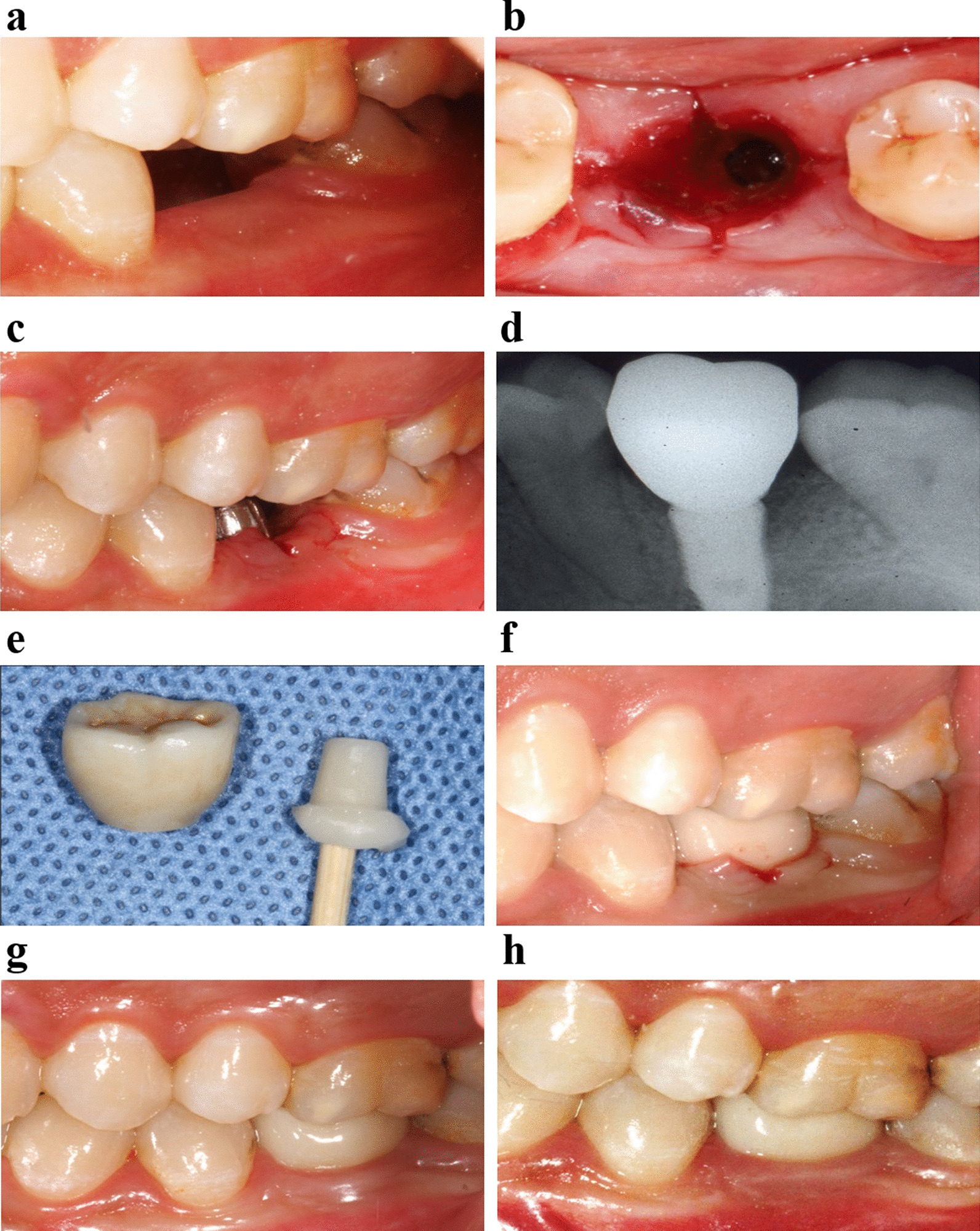
Clinic procedures for patients subjected to cross-shaped incision. **a** Buccal view of implant sites; **b** Cross-shaped incision was made across gingival sulcus after the disconnection of healing abutments; **c** Buccal view of the cross-shaped incision with definitive abutment; **d** X-ray was taken to make sure the abutment and crown were properly seated; **e** Customized resin abutment was made; **f** Buccal view of free gingival around implant after final restoration; **g** Buccal view of gingival papilla around implant 3 months after final restoration; **h** Buccal view of gingival papilla around implant 12 months final restoration

Two weeks later, the try-in was carried out after the fabrication of a definitive abutment and crown. The procedures were as follows: the patients were given 1 ml articaine for local infiltration anesthesia. The healing abutment was disconnected, and the cross-shaped incision was made at the buccal, lingual, mesial and distal aspects across the gingival sulcus using 12^#^ blade. The cross-shaped incisions went directly to the bone surface. The length of the cross-shaped incisions was 1–2 mm in keratinaized gingiva (Fig. 2a, b). X-ray was taken to make sure that the abutment and crown were properly seated after try-in of definitive abutments and crowns (Fig. 2c, d). The crown was cleaned after occlusal adjustment. Then screw of the abutment was tightened with a torque nearly 35 N cm using the screw driver connected to a torque wrench. Before bonding of the crown, exudation in gingival sulcus was stopped by cotton balls for about 1 h. Various methods were used to minimize excess cement extrusion into the peri-implant tissue. Customized abutment replica was made of acrylic resin before the cement. The crown was inserted onto the replica, permitting the extrusion of excess cement (Fig. 2e). The crown was placed onto the abutment (Fig. 2f). Dental floss was positioned mesial and distal surfaces of the prosthesis in order to remove the excessive cement after clotting. Antibiotic and anti-inflammatory drugs were not recommended. The patients were requested not to brush the surgical area in 24 h. Chlorhexidine mouthwash was routinely used to maintain good oral hygiene after surgery and crown placement.

### Clinical follow-up

**Fig. 3:**
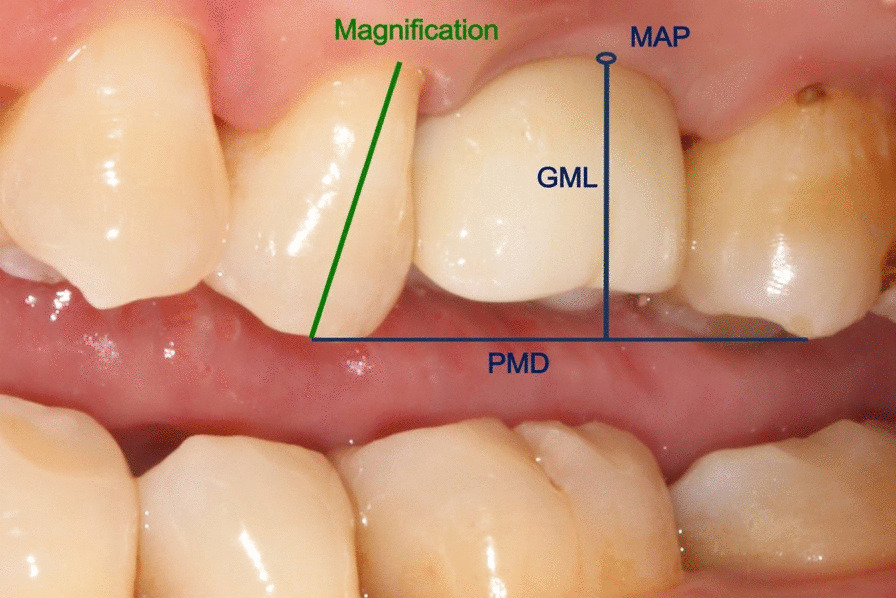
Schematic drawing illustrating the landmarks used for the measurement of gingival marginal level. MAP: the most apical point of the gingival margin at the buccal aspect of the crown; PMD: the line connecting the peak of the adjacent mesial and distal natural teeth (PMD); GML: the distance from MAP to PMD; Magnification: the length of the natural crown next to the implant supported prosthesis was recorded to correct any changes in magnification

All patients in this study accepted oral hygiene instruction at each visit. All patients were examined at 3-month and 12-month after final restoration (Fig. 2g, h) for the following examinations:

1. Presence/absence of papilla height was assessed visually according to the papilla index proposed by Jemt [14].
2. Modified Plaque Index (mPI): plaque accumulation around the marginal peri-implant tissue was assessed by the criteria of mPI [15].
3. Modified Sulcus Bleeding Index (mBI): the bleeding tendency of the marginal peri-implant tissue was evaluated using mBI [15].
4. Probing Depth (PD, mm): PD was assessed at the mid-buccal, mid-oral, mesial and distal aspects of the buccal surfaces of each implant with a standard periodontal probe, and final value was determined by the average of four aspects.
5. Gingival margin level (GML): gingival margin level was assessed by calculating the vertical distance between the most apical point of gingival margin at the buccal aspect of the crown and line connecting the peak of the adjacent mesial and distal natural teeth (PMD) [16]. The length of the natural crown next to the implant supported restoration was recorded to correct any changes in magnification (Fig. 3).

### Radiographic follow-up

**Fig. 4:**
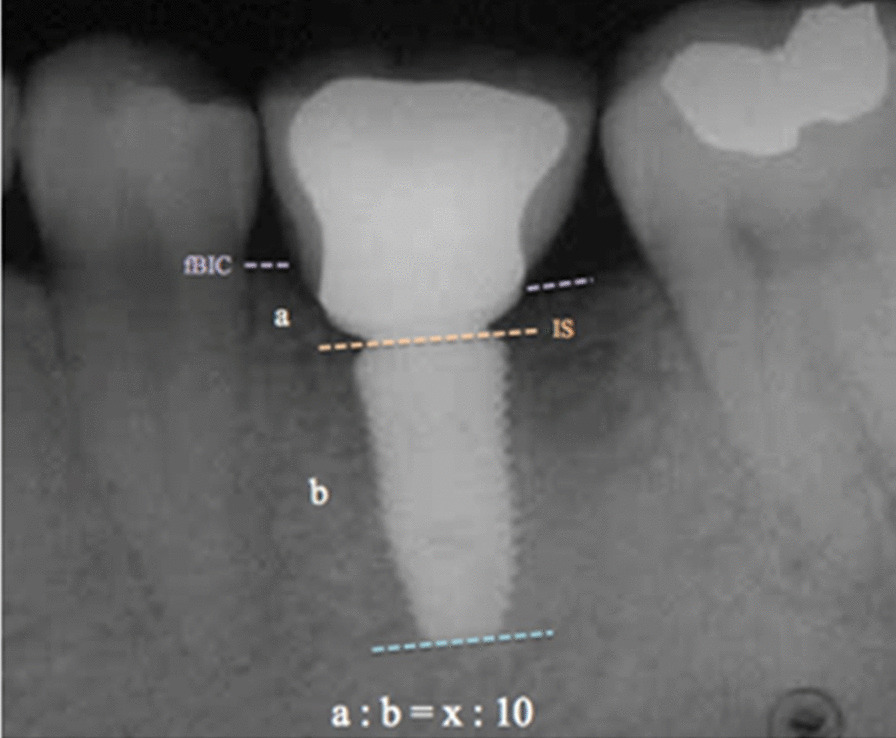
Schematic drawing illustrating the landmarks used for periapical radiographs measurement. IS: implant shoulder; fBIC: first bone-implant contact; **a** the vertical distance the first bone-implant contact to implant shoulder measured from radiograph. **b** Implant length. x (fBIC-IS): the real vertical distance the first bone-implant contact to implant shoulder

Periapical radiographs of the implant-supported crown was taken using the parallel photographing technique at each recall examination. To be specific, the landmarks of first bone-implant contact (fBIC) and implant shoulder (IS) were used for measurements. fBIC-IS was defined as the vertical distance the first bone-implant contact to implant shoulder, and the distance was assessed at the mesial and distal aspect of implant, respectively. When the marginal crestal bone was located coronal to the IS, a positive (+) value was given, where a negative (–) value when located apically to the IS, the value was deemed as zero when IS and fBIC coincided. The crestal bone level at the time of impression taking was regarded as baseline (Fig. 4). The known implant length was used for the calibration of dimensional distortion in the radiograph (the length of implant was 10 mm in Fig. 4).

### Statistical analysis

The statistical analysis was performed using GraphPad Prism 6.0 program. The data were presented as means ± standard deviations (SD), the differences between thin-gingiva group and thick-gingiva group were compared using the paired t test. A difference was considered significant if the P value was < 0.05.

## Results

During the study period, a total of 55 patients were included. Patients were grouped into 2 groups according to gingival phenotype. Detailed demographic data and sample characteristics were presented in Table 1. All patients were treated with implants placement in posterior areas. Three months after final restoration, all implants showed stable osseointegration with the absence of pain or inflammation, the absence of peri-implant radiolucency, and the absence of screw or crown loosening. There was no complain in both thick-gingiva group and thin-gingiva group.

**Table 1:** Demographic data of the patients

Demographics	Thin-gingiva	Thick-gingiva
Patients (n)	29	26
Age range (years)	24–58	21–62
Mean ± SD	40.14 ± 10.75	38.69 ± 11.06
Sex (n: femal/male)	13/16	15/11
Implant location		
(n: maxilla/madible)	18/11	14/12

Data were presented as means ± standard deviations (SD)

The Jemt parameters of soft tissue were listed in Table 2. In thick-gingiva group, both mesial and distal papilla filled more than half of the proximal space and in good harmony with the adjacent papillae 3 months after restoration; and the papilla height didn’t change significantly over one year. Although the score of papillae height in thin-gingiva group was lower than thick-gingiva group, there was no statistical difference between two groups (P > 0.05). From 3 to 12-month visit, the score of mPI and mBI decreased slightly in both thin-gingiva group and thick-gingiva group, but the change didn’t show significant difference (P > 0.05). At 3-month visit, the PD in thin-gingiva group and thick-gingiva group was 2.16 ± 0.42 mm and 2.18 ± 0.41 mm, respectively, which remained almost the same from 3 to 12-month visit in both groups (P > 0.05).

**Table 2:** Clinical Jemt parameters of soft tissue from 3 to 12 months

Parameters	Follow-up (months)	Thin-gingiva	Thick-gingiva	*P* value
Mesial papilla	3 12	2.62 ± 0.62 2.41 ± 0.63	2.77 ± 0.66 2.54 ± 0.58	0.39 0.45
Distal papilla	3 12	2.52 ± 0.64 2.31 ± 0.66	2.69 ± 0.68 2.42 ± 0.58	0.33 0.51
mPI (mm)	3 12	0.89 ± 0.79 1.04 ± 0.84	1.04 ± 0.82 0.88 ± 0.65	0.51 0.47
mBI(mm)	3 12	0.61 ± 0.74 0.75 ± 0.88	0.77 ± 0.95 0.65 ± 0.80	0.49 0.68
PD (mm)	3 12	2.16 ± 0.42 1.94 ± 0.47	2.18 ± 0.41 2.07 ± 0.45	0.81 0.29
GML(mm)	Baseline	7.59 ± 1.24	7.17 ± 1.26	–
	3	8.23 ± 1.26^△^	7.53 ± 1.29	0.008*
	12	9.01 ± 1.30^△^	8.35 ± 1.48	0.04*

Data were presented as means ± standard deviations (SD)

*Significant difference (P < 0.05) between thin-gingiva group and thick-gingiva group;

^△^Significant difference (P < 0.05) between 3 months or 12 months and baseline

**Fig. 5:**
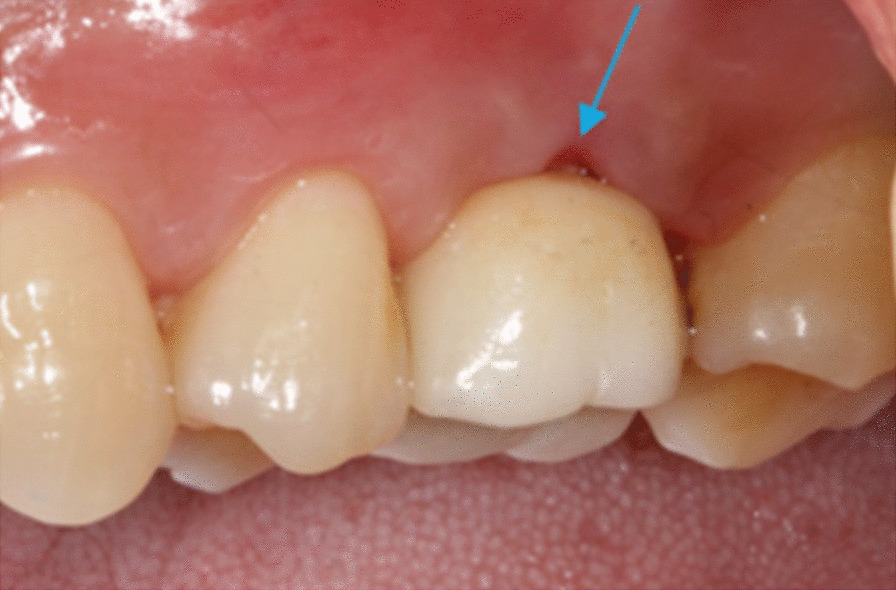
Recession of marginal gingiva was found in the middle of buccal aspect around crown 3 months after cement

The GML on the buccal aspect of the crown was assessed by the distance from TBF to PMD. Table 2 showed that at 3-month or 12-month visit, GML in thin-gingiva group increased significantly compared to the baseline (P < 0.05), higher than the GML in patients with thick-gingival phenotype. In thick-gingiva group, the GML remained stable during the whole follow-up period, there has no obvious change about the GML from baseline to 12-month visit (P > 0.05). According to clinical examinations, the recession of marginal gingiva of buccal aspect of the crown was found in 5 patients with thin-gingiva during the follow-up period (Fig. 5).

The change of peri-implant hard tissue was shown in Table 3. Implant was inserted into the maxillae or mandible located coronal to the IS, and there has no difference in fBIC-IS between thin-gingiva group and thick-gingiva group. The mean fBIC-IS in thin-gingiva group decreased from 0.40 ± 0.95 at 3-month visit to -0.02 ± 1.01 at 12-month visit; and the value in thick-gingiva group decreased from 0.29 ± 0.59 in 3-month visit to -0.11 ± 0.63 at 12-month visit (P < 0.05). Although both thin-gingiva group and thick-gingiva group showed slight marginal bone resorption, there has no significant difference between thin-gingiva group and thick-gingiva group (P > 0.05).

**Table 3:** Clinical parameters of hard tissue from 3 to 12 months

Parameters	Follow-up (month)	Thin-gingiva	Thick-gingiva	*P* value
Mesial fBIC-IS	Baseline	1.01 ± 0.82	0.72 ± 0.69	0.93
	3	0.44 ± 1.12^△^	0.26 ± 0.76^△^	0.67
	12	0.03 ± 1.31^△^	− 0.02 ± 0.74^△^	0.91
Distal fBIC-IS	Baseline	0.81 ± 0.82	0.71 ± 0.69^△^	0.81
	3	0.33 ± 0.92^△^	0.20 ± 0.74^△^	0.58
	12	− 0.07 ± 0.98^△^	− 0.20 ± 0.78^△^	0.82
Mean fBIC-IS ((M + D)/2)	Baseline	0.91 ± 0.70	0.72 ± 0.62^△^	0.93
	3	0.40 ± 0.95^△^	0.29 ± 0.59^△^	0.77
	12	− 0.02 ± 1.01^△^	− 0.11 ± 0.63^△^	0.82

Data were presented as means ± standard deviations (SD)

^△^Significant difference (P < 0.05) between 3 months or 12 months and baseline

## Discussion

The purpose of this study was to evaluate clinical and radiographic manifestations of a cross-shaped incision technique for thick-gingiva and thin-gingiva patients treated with implant-supported fixed prosthesis. Our study demonstrated that papillae filled more than half of the proximal space, in good harmony with the adjacent papillae in both thin-gingiva and thick-gingiva groups. In our study, zirconia crowns were fabricated and cemented to abutments with the lowest gingival height. Welander et al. demonstrated an apical shift of the barrier epithelium and the marginal bone around AuPt-alloy, where the soft tissue dimensions remained stable around Ti and ZrO_2_ abutments [17]. Kajiwara et al. demonstrated that greater blood flow was detected around zirconia abutment group compared to the titanium abutment or cast-to-abutment in free gingiva [18]. And we proposed that zirconia crowns may promote microcirculatory dynamics in soft tissue and be beneficial for the bone remodeling around implant.

Moreover, securing a rich blood flow in soft tissues around implants is considered to be advantageous for the maintenance of immune function [18], which was reflected by the low score of mPI and mBI in this study. All patients in this study accepted oral hygiene instruction at each visit, so PD in both thick-gingiva and thin-gingiva groups remained healthy. The values of PD even decreased slightly from 3 to 12-month after restoration, perhaps due to the emphasized instruction of oral hygiene at each visit.

The importance of possessing an adequate width and thickness of keratinized mucosa seems to be crucial both for natural teeth and dental implants. A deficiency of (or minimal) keratinized mucosa around implants has been shown to hinder patient oral hygiene, leading to soft tissue inflammation, mucosal recession, and attachment loss [19]. Having at least 2 mm of keratinized tissue width had protective effect on peri-implant health, and implants with < 2 mm of keratinized tissue width were more prone to develop peri-implant biologic complications [20]. The patients included in this study had at least 2 mm of keratinized tissue width around the implant. However, the recession of marginal gingiva was detected in patients with thin-gingival phenotype. Gingival phenotype, thin or thick, may affect the dimension of the periodontal tissue. A thick phenotype is prone to pocket formation, while a thin phenotype is prone to gingival recession following mechanical or surgical manipulation [6]. Different from natural teeth, supracrestal fibers (gingivo-dental and transseptal fibers) was not in the gingival tissue surrounding the implant abutment. Furthermore, the absence of blood vessel branches associated with the periodontal ligament results in restricted blood supply to the peri-implant mucosa. The pressure between the restoration and gingiva typically causes ischemia [7]. Therefore, the peri-implant mucosa can also be defined as “scar-like” tissues. The gingival with thin phenotype around implant is easier to shrink following surgery, which may be the reason of gingival recession in the middle of buccal aspect in patients with cross-shaped incision. Compared with thin-gingiva group, GML did not change from baseline to the 12-month visit in thick-gingiva group, indicating consistent stability of the gingival margin after the cross-shaped incision of gingival sulcus around the crown in patients with thick-gingival phenotype.

Marginal crestal bone in both thin-gingiva and thick-gingiva groups was located coronal to the IS at the beginning, then decreased slightly during the following year, in accordance with the results of other studies [21, 22]. The stability of marginal bone may be due to the protection of soft tissue barrier, which serves as a protective seal for the adjacent periodontium [23]. Furthermore, “platform switching” was used in all the implants, which may result in less marginal bone resorption [24, 25]. In turn, the underlying bone provides the support of gingival tissue [6, 26].

Based on these results, the zirconia crown is beneficial for the reconstruction of gingival papillae by promoting microcirculatory dynamics in soft tissue around implant. The gingival margin remained stable in patients with thick phenotype gingiva after the cross-shaped incision of gingival sulcus around the crown. The cross-shaped incision has several advantages. It is visible to check the proper placement of crowns and easy to clean excessive cement with the slight lift of incised gingiva. The limitation for cross-shaped incision is that the recession of marginal gingiva where the incision is made may happen in thin-gingiva group. The patients included must have at least 2 mm of keratinized tissue width around the implant. The lack of negative controls was another limitation of this study.

## Conclusion

The cross-shaped incision may be applied to reconstruct gingival papillae and avoid the gingival recession in patients with thick-gingiva phenotype. For the patients with thin-gingival phenotype, a modified method aimed to reconstruct gingival papillae and avoid the gingival recession need further study.

## Data Availability

The datasets used and/or analysed during the current study are available from the corresponding author on reasonable request.

## Abbreviations

fBIC: First bone-implant contact
GML: Gingival margin level
IS: Implant shoulder
mBI: Modified Sulcus Bleeding Index
mPI: Modified Plaque Index
PD: Probing Depth
VIP-CT: Vascularized interposition periosteal-connective tissue
